# Advancing Kir4.2 Channel Ligand Identification through Collision-Induced Affinity Selection Mass Spectrometry

**DOI:** 10.1021/acschembio.3c00781

**Published:** 2024-03-07

**Authors:** Yushu Gu, Miaomiao Liu, Linlin Ma, Ronald J. Quinn

**Affiliations:** † Griffith Institute for Drug Discovery, Griffith University, Brisbane, Queensland 4111, Australia; ‡ School of Environment and Science, Griffith University, Brisbane, Queensland 4111, Australia

## Abstract

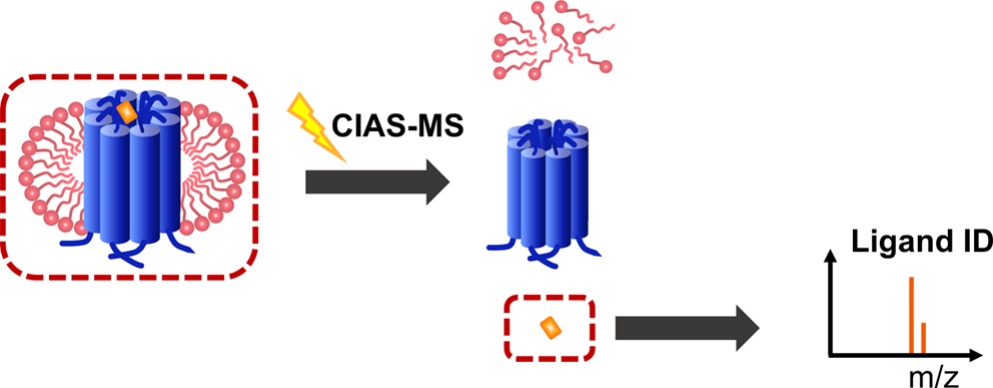

The inwardly rectifying potassium Kir4.2 channel plays a crucial role in regulating membrane potentials and maintaining potassium homeostasis. Kir4.2 has been implicated in various physiological processes, including insulin secretion, gastric acid regulation, and the pathogenesis of central nervous system diseases. Despite its significance, the number of identified ligands for Kir4.2 remains limited. In this study, we established a method to directly observe ligands avoiding a requirement to observe the high-mass ligand-membrane protein-detergent complexes. This method used collision-induced affinity selection mass spectrometry (CIAS-MS) to identify ligands dissociated from the Kir4.2 channel-detergent complex. The CIAS-MS approach integrated all stages of affinity selection within the mass spectrometer, offering advantages in terms of time efficiency and cost-effectiveness. Additionally, we explored the effect of collisional voltage ramps on the dissociation behavior of the ligand and the ligand at different concentrations, demonstrating dose dependency.

Inwardly rectifying potassium (Kir) channels, belonging to the superfamily of potassium (K^+^) ion channels, play essential roles in maintaining resting membrane potential, modulating the duration of action potentials, and regulating K^+^ homeostasis.^1,2^ The voltage-independent Kir channels facilitate the movement of K^+^ ions in the inward direction rather than in the outward direction at the same driving force of the opposite direction.^1^ The predicted topology and 3D structures of Kir channels, using Kir4.2 as the model, suggest that each monomer channel protein comprises two transmembrane helices accompanied by cytoplasmic NH_2_ and COOH termini, as well as an intramembrane pore-forming region (Figure 1A,B).^1,3,4^ There are seven different subfamilies in Kir channels (Kir1.x-Kir7.x) that can be categorized into four functional groups: K^+^ transport channels (Kir1.x; Kir4.x; Kir5.x, and Kir7.x), classical Kir channels (Kir2.x), G protein-coupled Kir channels (GIRK, Kir3.x), and ATP-sensitive K^+^ channels (Kir6.x).^1,5^

**Figure 1:**
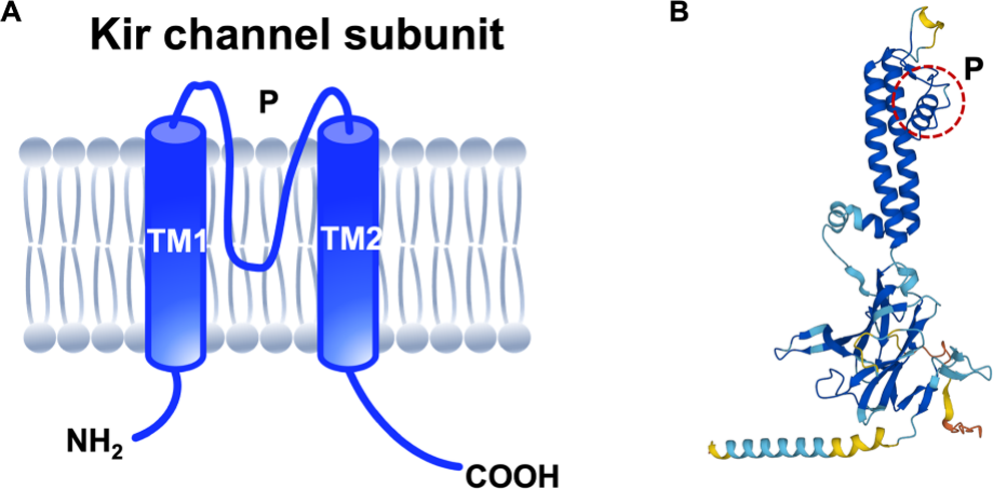
Predicted topology and 3D structures of Kir4.2 channel. (A) Topology structure of the Kir channel subunit, which includes two transmembrane regions (TM1 and TM2), a pore-forming loop (P), and cytosolic NH2 and COOH termini. (B) Predicted 3D structures of Kir4.2 with pore region highlighted in red circle. Dark blue: very high (pLDDT > 90); light blue: confident (90 > pLDDT > 70); yellow: low (70 > pLDDT > 50), and orange: very low (pLDDT < 50). pLDDT—per-residue confidence score.

The inwardly rectifying potassium channel, subfamily J, member 15 (*KCNJ15*) gene, which encodes Kir4.2 channel, has been identified to have an inhibitory effect on insulin secretion in pancreatic β cells by maintaining the resting membrane potential of these cells, thereby inhibiting depolarization processes involved in insulin release.^6−8^ Kir4.2 is the most prominently expressed among all K^+^ channels in the stomach, playing a pivotal role in regulating gastric acid secretion.^9,10^ The *KCNJ15* gene resides within the Down syndrome chromosome region 1 on chromosome 21 and has been implicated in the pathogenesis of Down syndrome.^11,12^ Previous studies have also suggested that its involvement is not limited to metabolic disorders and Down syndrome, but also extends to the pathogenesis of several central nervous system diseases.^13^ It has been implicated as a potential biological target for Parkinson’s disease, Alzheimer’s disease, and epilepsy, suggesting that it is involved more broadly in the pathogenesis of central nervous system disorders.^13−15^ Despite the significance of Kir4.2 as a molecular target, the number of its identified ligands remains limited. Only a few compounds, including polymyxins B and VU0134992, have been reported to interact with Kir4.2.^16,17^ However, there are currently no specific Kir4.2 modulators available, making it challenging to selectively activate or inhibit the channel functions for mechanistic studies. This highlights the need for developing innovative screening techniques that could facilitate the identification of more ligands for this ion channel.

Investigation of membrane proteins poses challenges owing to their amphipathic nature, low natural abundance, and difficulties in overexpression and purification.^18^ Native mass spectrometry (native MS) has emerged as a robust platform for investigating membrane proteins, using collisional activation to liberate membrane proteins from detergent micelles.^19−24^ Native MS has proven valuable in characterizing protein–lipid interactions.^25−31^ Within the context of Kir channels, lipids binding to Kir3.2 and Kir3.4 have been reported.^28−31^ The selectivity of Kir3.2 toward various phospholipids was investigated, revealing that Kir3.2 exhibits a preference for phosphatidylinositide (PIP) isoforms, particularly for the phosphatidylinositol 4,5-bisphosphate (PI(4,5)P_2_) headgroup, compared to other phospholipids.^29,30^ Kir3.4 also displayed differences in lipid binding selectivity, with weaker interactions with PIPs compared to that of Kir3.2.^31^ The binding thermodynamics between Kir3.2 and lipids showed that the interaction between Kir3.2 and specific PIPs is highly influenced by entropy.^28^

Identification of ligands bound to membrane protein in MS has been demonstrated using Nativeomics, which combines native MS and small-molecule fragmentation, enabling the detection of bound molecules ejected after native MS.^19^ In Nativeomics, the protein–ligand assembly is first released from the detergent micelle encapsulating it, and the assembly is then isolated and selected to be dissociated into proteoforms and ligands.^19^ In this method, the mass spectrometer must be capable of detecting both intact protein–ligand complexes at the high mass-to-charge *(m/z)* range and fragmented ligand at the lower *m*/*z* range.^19,32^

We have recently reported the application of collision-induced affinity selection MS (CIAS-MS) (Figure 2A).^33^ General affinity selection mass spectrometry (AS-MS) techniques require several preparation steps prior to the mass spectrometric identification of a ligand (Figure 2B).^34−36^ CIAS-MS eliminates the need for prescreening removal of unbound compounds by conducting all procedures within the mass spectrometry itself. Briefly, CIAS-MS employs a quadrupole for mass selection, trapping the protein–ligand complexes while removing unbound molecules. Collision-induced dissociation (CID) is applied to dissociate the protein–ligand complex; the dissociated protein and ligand are then transferred to the ion guide featuring a low time of flight, enabling only the small-sized ligand to be transferred into the mass analyzer for detection (Figure 2A).^33^ We have shown that the feasibility of CIAS-MS in the screening of SARS-CoV-2 nonstructural protein 9 (nsp9) with a mixture of compounds.^33^

**Figure 2:**
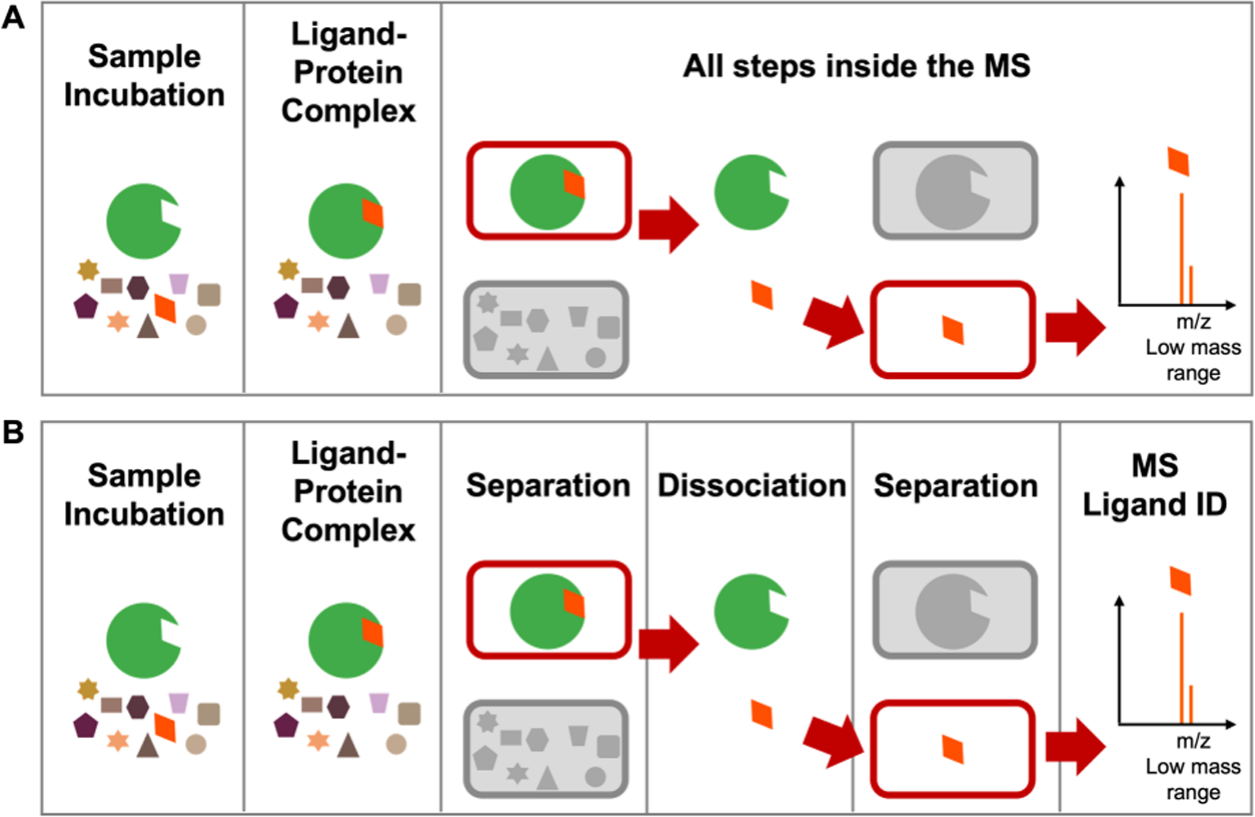
Comparison of CIAS-MS and AS-MS. (A) CIAS-MS involves the direct injection of the sample mixture into the mass spectrometer, where all of the necessary steps are performed internally. (B) AS-MS begins with an incubation step to allow for the formation of protein–ligand complexes, followed by the separation of these complexes from unbound compounds. The bound ligand is then dissociated, and a subsequent separation step is performed to capture the low-molecular-weight (MW) ligand. Finally, the ligand is injected into the mass spectrometer for analysis.

In this study, we explored CIAS-MS for the Kir4.2 channel and its agonist polymyxin B. Polymyxin B is a polypeptide antibiotic derived from the soil organism *Bacillus polymyxa* (Figure 3).^37^ It is commonly used in clinical settings to treat minor skin infections caused by Gram-negative bacterial species, including *Pseudomonas*.^38^ A recent study revealed that polymyxin B1 activates the Kir4.2 channel, leading to influx of potassium and depolarization of the cell membrane.^16^ Molecular modeling suggested that polymyxin B binds spontaneously to the extracellular region of Kir4.2 within a lipid bilayer.^16^ We investigated the binding of polymyxin B to the Kir4.2 channel using CIAS-MS. To demonstrate the specificity of this method, the interaction between the Kir4.2 channel and polymyxin B was examined in a pooled library containing 100 compounds. Kir4.2 was also screened against a natural product library containing 2000 compounds. Subsequently, we explored and compared the impact of various collisional voltages on the dissociation behavior of the binding ligands.

**Figure 3:**
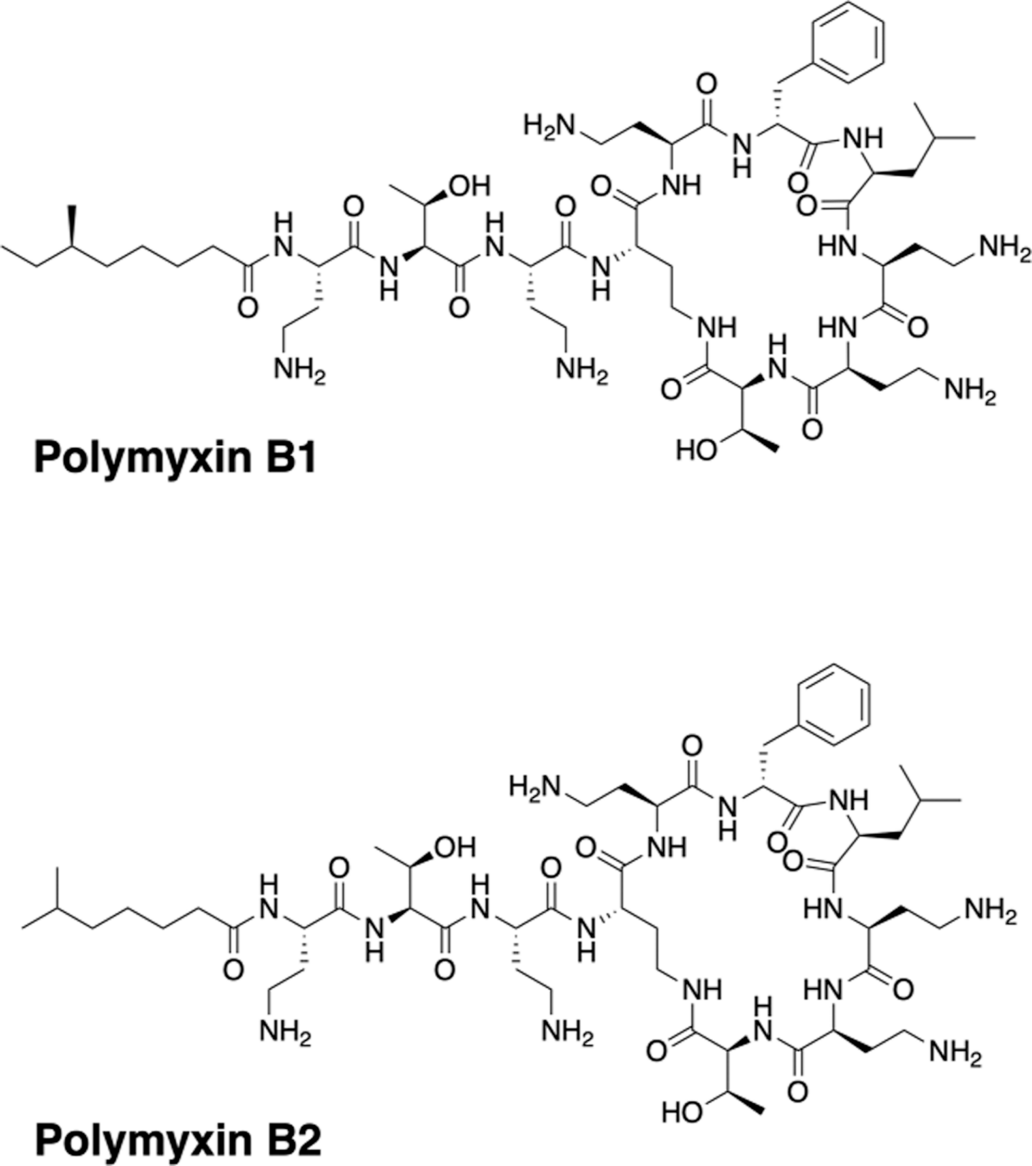
Chemical structures of polymyxins B1 and polymyxin B2.

## Results and Discussion

### Confirmation of Kir4.2 Expression

**Figure 4:**
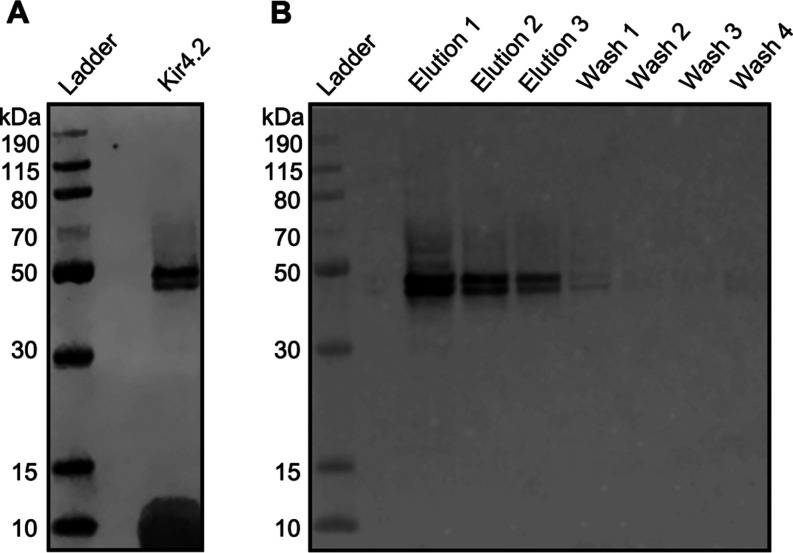
Western blot analysis with a Thermo Fisher PageRuler prestained protein ladder confirmed the expression, purification, and solubilization of Kir4.2. (A) Solubilized Kir4.2 after second ultracentrifugation step to remove any insolubilized material. (B) IMAC purified Kir4.2 was fully eluted in the three elution steps, without passing through to the wash steps.

The Kir4.2-expressing construct was generated using the pEF6/V5-His vector and expressed in Expi293F cells. Following expression, the protein was solubilized in dodecylmaltoside (DDM) and purified using immobilized metal affinity chromatography (IMAC) with four wash steps using wash buffer containing 25 mM imidazole and 0.2% DDM, and three elution steps with elution buffer composed of 400 mM imidazole and 0.05% DDM. To validate the successful overexpression of Kir4.2 channel proteins in the Expi293F cells and their enrichment after the purification step in eluted samples that were subjected to the subsequent MS studies, Western blot analysis was employed with an anti-V5 tag antibody. As shown in Figure 4A, Kir4.2 proteins were effectively solubilized during the second ultracentrifuge step, which removed unsolubilized material. The eluted Kir4.2 proteins presented as two bands, approximately 43 and 45 kDa, on the blot. These bands likely represent fully and partially glycosylated forms of the protein, respectively, which is consistent with previous reports indicating the presence of a glycosylation site in Kir4.2.^39,40^ Protein concentrations noticeably decreased across successive elution cycles with minimal detection in later washes, indicating a high level of purification efficiency (Figure 4B).

### CIAS-MS of Kir4.2 Binding to Its Agonist Polymyxin B

Previous studies have demonstrated that the protein–DDM micelle complex can be transferred into the gas phase while maintaining binding to ligand within the complex.^41^ Typically, the liberation of membrane protein is achieved by disrupting the detergent micelle using activation energy within the MS.^20,42^ The overall activation energy which can be tuned in a Fourier transform-ion cyclotron resonance (FT-ICR) mass spectrometer is skimmer 1 located at the end of funnel 2 and CID of the collision cell (analogous to cone voltage and trap voltage in a quadrupole time-of-flight (Q-ToF) platform).^42^ The manipulation of the activation settings in the FT-ICR platform has been investigated extensively, with the resulting impact compared to the Q-ToF platform previously.^42^ The findings demonstrated that, while FT-ICR generally produces lower protein-to-detergent ratios under full MS mode compared to Q-ToF instruments, the high resolution of the FT-ICR aids the analysis of membrane protein samples with low MW modifications.^42^

**Figure 5:**
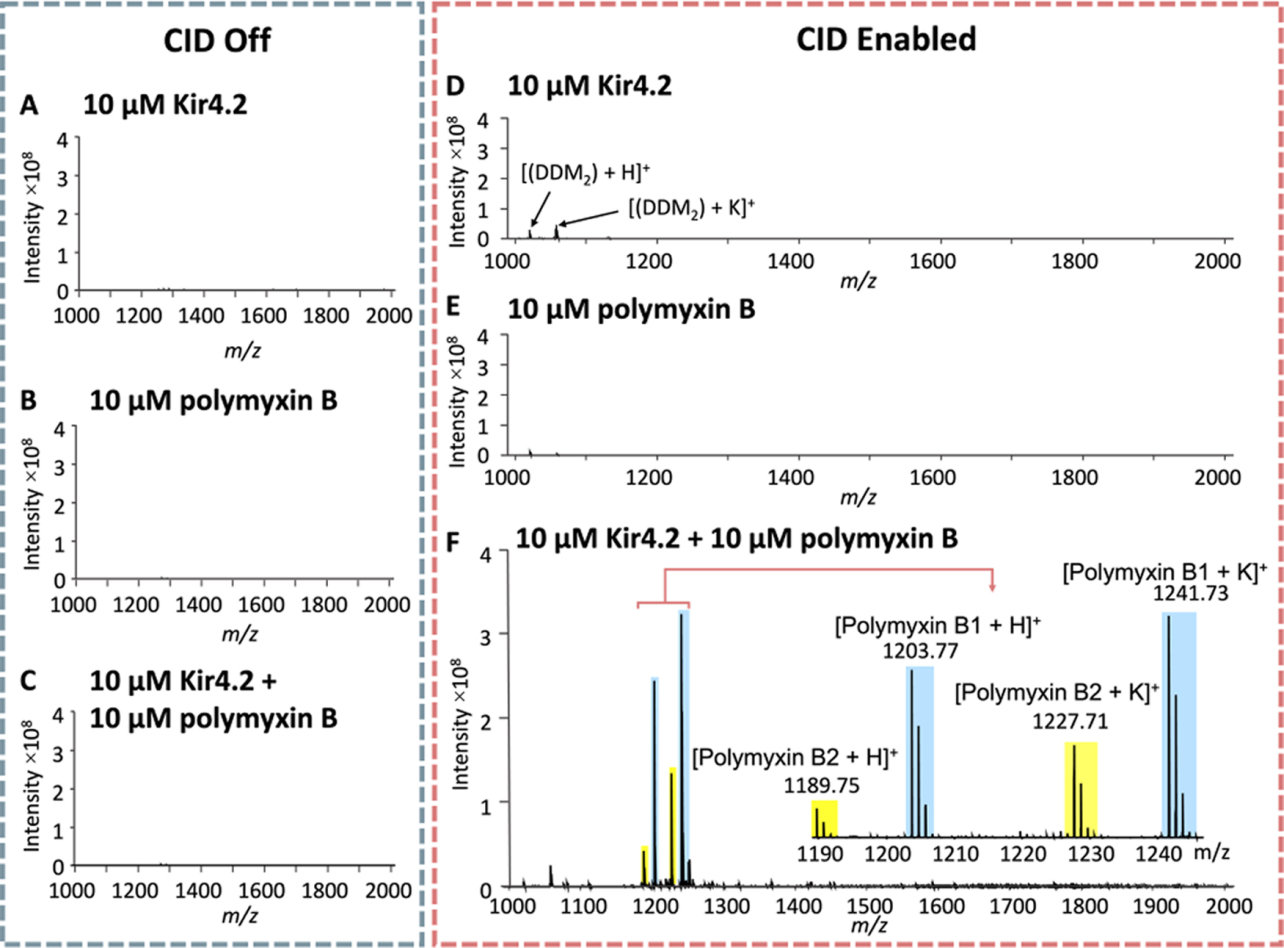
CIAS-MS of Kir4.2. Control experiment with CID turned off: (A) Kir4.2 (10 μM) alone; (B) Polymyxin B (10 μM) alone; (C) Kir4.2 (10 μM) incubated with polymyxin B (10 μM). CIAS-MS with CID enabled: (D) Kir4.2 alone (10 μM); (E) Polymyxin B alone (10 μM) and (F) Kir4.2 (10 μM) incubated with polymyxin B (10 μM).

We first successfully detected the dissociation of polymyxin B from the Kir4.2-polymyxin B complex. This began with the electrospray ionization of the complex within the detergent micelles and transmission into the MS. Skimmer 1 was adjusted to facilitate ion transmission and aid in in-source dissociation. The quadrupole was configured to capture the protein–ligand complexes above *m*/*z* 2500 while small unbound molecules. The complexes were transferred to the collision cell, where a CID voltage was applied to allow ejection of the complexes from the detergent micelle and induce dissociation of the bound ligand. Control experiments were conducted with Kir4.2 (10 μM) alone or polymyxin B (10 μM) alone, both without (Figure 5A,B) and with (Figure 5D,E) CID enabled. The spectra revealed no ions corresponding to the MW of polymyxin B in the mass range of 500–2500 *m*/*z*. This indicated that the mass selection settings in the quadrupole effectively excluded any unbound ligands. In the presence of CID activation, the CIAS-MS spectrum of Kir4.2 alone produced peaks corresponding to fragmented DDM molecules with a hydrogen adduct and a potassium adduct (Figure 5D).

When 10 μM Kir4.2 incubated with 10 μM polymyxin B (composed of approximately 90% polymyxin B1 and 10% polymyxin B2) was injected into the MS, no ions were detected without CID activation (Figure 5C). Upon enabling CID, the major signal observed for polymyxin B1 was *m*/*z* 1241.73, which represented a potassium adduct and the major signal observed for polymyxin B2 was *m*/*z* 1227.71, corresponding to a polymyxin B2 potassium adduct. [M + H]^+^ species were also detected for both polymyxin B1 and B2, at *m*/*z* 1203.77 and 1189.75, respectively (Figure 5F). In contrast, the base peaks of polymyxins B1 and B2 were observed as [M + H]^+^ species under positive-ion mode, with 9 and 34% potassium adducts, respectively, suggesting the generation of adduct ions inside the collision cell (Figure S1). These findings demonstrate that CIAS-MS using FT-ICR can transfer Kir4.2-polymyxin B complex into the gas phase, remove unbound ligands with the quadrupole mass analyzer, and dissociate the ligand from the protein–ligand–detergent complex.

### CIAS-MS Spiking Experiment for Kir4.2

**Figure 6:**
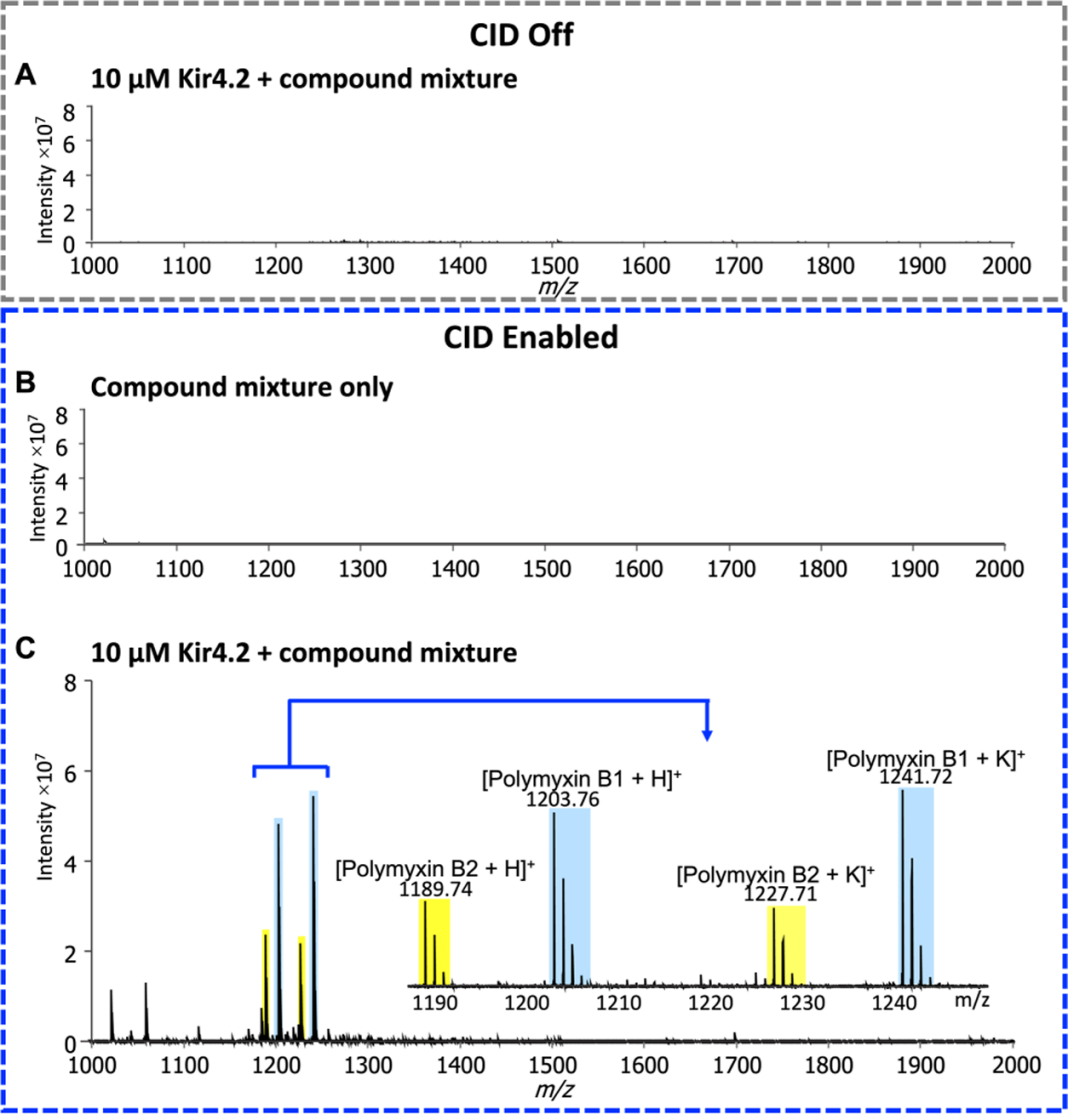
CIAS-MS of 10 μM Kir4.2 incubated with a compound mixture containing 100 compounds (each at a concentration of 10 μM) including polymyxin B. (A) Kir4.2 (10 μM) incubated with a compound mixture without CID application. (B) Compound mixture only with CID enabled. (C) Kir4.2 (10 μM) incubated with a compound pool with CID enabled.

To assess the applicability of CIAS-MS in detecting specific interaction between a membrane protein and its ligand with pooled compound libraries, Kir4.2 at a concentration of 10 μM was screened against a natural product library pool comprising 100 molecules at a concentration of 10 μM each spiked with polymyxin B. Without CID activation, no ions were observed (Figure 6A). A control experiment was also conducted by injecting the compound library mixture alone with CID enabled with no ions detected in the mass range (Figure 6B). Upon enabling CID, four ion signals were detected at *m*/*z* 1203.76, 1241.72, 1189.74, and 1227.71, which corresponded to polymyxin B1 with a hydrogen adduct and a potassium adduct, polymyxin B2 with a hydrogen adduct and a potassium adduct, respectively (Figure 6C). We have previously reported the capability of CIAS-MS in detecting binding ligands to a soluble protein, nsp9, in a pooled compound library of 9.^33^ Here, we show that CIAS-MS can identify the Kir4.2 agonist polymyxin B from a complex mixture of 100 compounds incubated with the ion channel that is solubilized in a detergent micelle.

### CIAS-MS Library Screening for Kir4.2

A natural product library comprising 2000 compounds, with each pool containing 100 compounds, each compound at a concentration of 10 μM (pools A-V), was screened against Kir4.2. Control experiments were conducted by subjecting each library pool alone to CID activation, ensuring that the current CIAS-MS condition sufficiently excluded any unbound molecules (control experiment for the active pool can be found in Figure S2).

**Figure 7:**
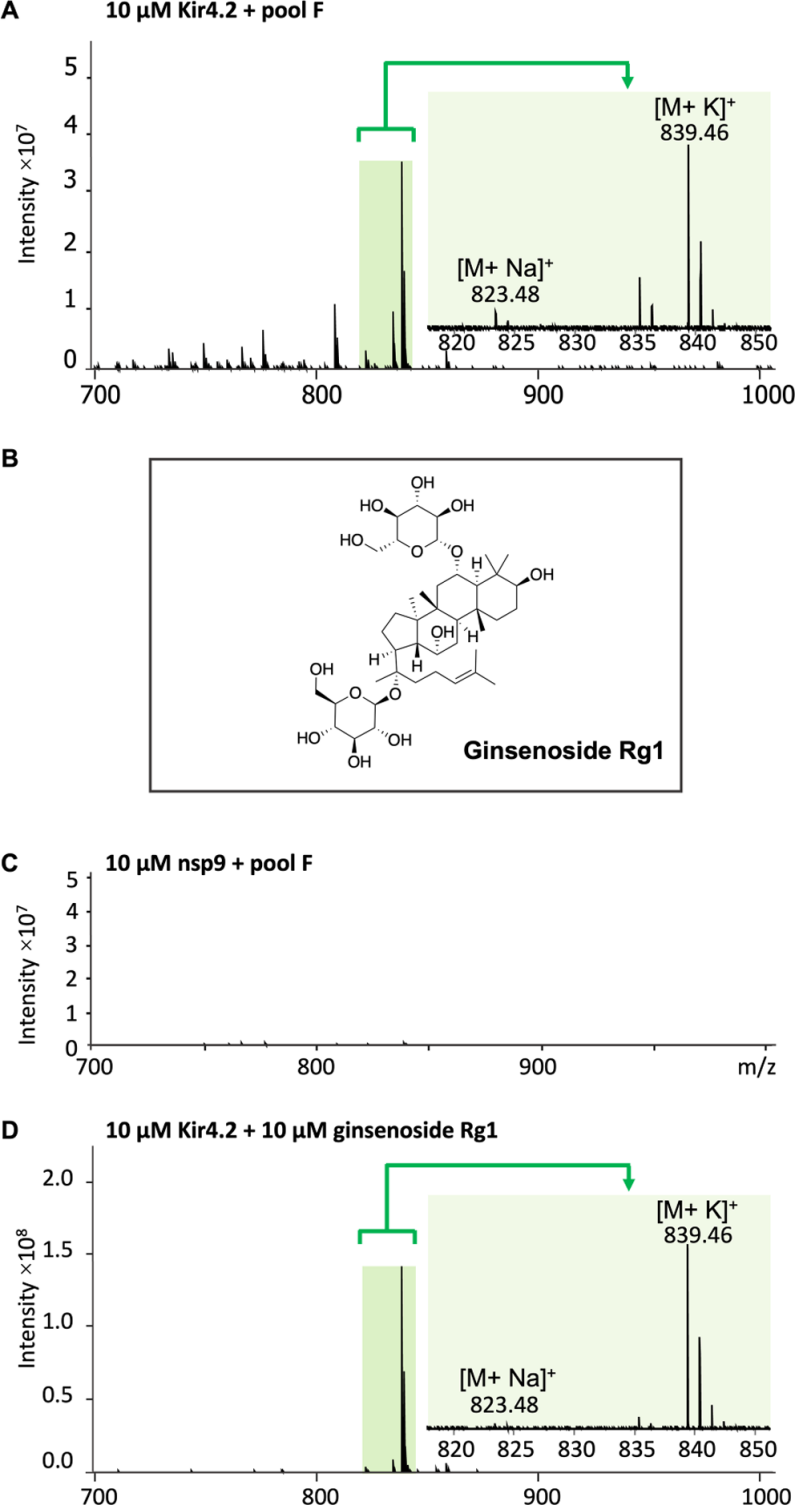
CIAS-MS under CID activation of (A) 10 μM Kir4.2 incubated with pool F (each compound at a concentration of 10 μM). (B) Chemical structure of ginsenoside Rg1. (C) 10 μM nsp9 incubated with pool F (each compound at a concentration of 10 μM). (D) 10 μM Kir4.2 incubated with ginsenoside Rg1 (10 μM).

In pool F, upon CID activation, multiple ion signals were observed, with the most prominent signals at *m*/*z* 839.46 and 823.48 (Figure 7A). The compound was identified as ginsenoside Rg1, with a potassium adduct as the base peak and a 9% sodium adduct (Figure 7B). Pool F was then incubated with 10 μM nsp9 protein, which had been employed to demonstrate the CIAS-MS capability in our previous study.^33^ Following the injection of this sample into the ESI source with CID enabled, no ions corresponding to the molecular weight of any compounds in the pool were detected. This suggests that no protein–ligand complexes were formed between nsp9 and the compounds in pool F (Figure 7C). This result was further confirmed by native MS analysis of nsp9 and pool F, which revealed the absence of binding interactions between nsp9 and the pooled compounds, particularly with ginsenoside Rg1 (Figure S3).

Next, CIAS-MS was employed to validate the binding of the pure ginsenoside Rg1 to Kir4.2. A control experiment of ginsenoside Rg1 alone under CID activation showed no ions in the mass range (Figure S2). After injecting the Kir4.2 and ginsenoside Rg1 mixture was injected, with CID enabled, two signals were detected at *m*/*z* 839.46 and 823.48, corresponding to ginsenoside Rg1 as the potassium adduct (base peak) along with a 3% sodium adduct (Figure 7D). Under positive-mode ESI conditions, ginsenoside Rg1 alone was observed as a base peak of *m*/*z* 839.45 (potassium adduct) and a 5% hydrogen adduct *m*/*z* 801.50 (Figure S4). The ginsenoside Rg1 hydrogen adduct species was not observed in the CIAS-MS of the ginsenoside Rg1-Kir4.2 complex under CID activation, suggesting that adduct ions were produced in the CID cell. Ginsenoside Rg1 is one of the main active components in ginseng with reported pharmacological activities such as anti-inflammatory and neuroprotective effects.^43−45^ Several studies have demonstrated the potential of ginsenoside Rg1 in the treatment of neurological disorders, including Parkinson’s disease, cerebral ischemia and reperfusion injury, and Alzheimer’s disease.^46−51^ Notably, ginsenoside Rg1 has been shown to reduce hippocampal Kir4.1 expression, a protein that has been implicated in several neurological diseases and possessing both structural and functional similarities to Kir4.2.^40,52−58^

### Investigation into Ligand Dissociation Behavior with CIAS-MS Voltage Ramping

Various MS-based techniques have been employed to determine the binding affinities of ligands, typically involving titration, or melting curve experiments.^59−63^ In titrations, the concentration of the ligand for the target protein is systematically altered, allowing for the measurement of changes in the equilibrium between the complex and the binding ligand. Melting curve experiments apply temperature ramps and monitor shifts in the equilibrium. In both systems, these shifts are then compared to the independent variable, and the binding affinity is calculated based on the resulting curve. Besides these two classical methods, laser-induced liquid bead ion desorption (LILBID) mass spectrometry has also been used in the quantitative evaluation of binding affinity.^64^ By modulating the laser energy directed at the sample droplets, the extent of complex dissociation can be altered.^64^ This allows for the determination of binding affinity based on the observed dissociation of the complexes. By manipulation of the energy transfer in this approach, dissociation curves like those obtained in melting curve experiments can be generated. Instead of adjusting the temperature, the energy input into the system is altered, leading to the dissociation of the complexes.

Notably, in CIAS-MS, the CID voltage applied to dissociate binding ligands from protein–ligand complexes can be manipulated. The amount of energy applied affects the dissociation of the ligand detected in the MS. Complexes stabilized by weak intermolecular interactions typically display low stabilities and are prone to dissociation.^65,66^ Similar to the concept of laser energy transfer in LILBID-MS, ligands with a higher binding affinity require greater CID energy to dissociate from the complex. Consequently, ligands with a strong binding affinity tend to dissociate relatively slowly as the voltage increases, and their dissociation persists even at higher voltages. Additionally, a higher ligand concentration typically leads to the formation of more protein–ligand complexes compared to a lower concentration. This increase in the abundance of complexes is expected to result in more dissociated ligands when collisional energy is applied.

Here, we investigated and compared the gas-phase dissociation behavior of the binders for Kir4.2. Samples containing protein–ligand mixtures were subjected to a CID voltage ramping. Each sample experienced a total of 21 voltage increments, ranging from 0 to 100 V, with intervals of 5 V, while all other parameters remained constant. The results were then interpreted through a CID voltage ramping dissociation curve, which plotted the signal-to-noise (S/N) ratio against the applied CID voltage. Data prior to plateau were included in the curve with values normalized based on the initial voltage at which bound ligand dissociation commenced. First, voltage ramping was conducted for Kir4.2 incubated with 10 μM polymyxin B exclusively. This data set was used to compare with three other voltage ramping data sets under the same ramping condition generated from (1) Kir4.2 incubated with 10 μM polymyxin B spiked into a pool of 100 compounds; (2) Kir4.2 incubated with a 10-fold higher concentration of polymyxin B (100 μM); and (3) Kir4.2 incubated with 10 μM ginsenoside Rg1.

**Figure 8:**
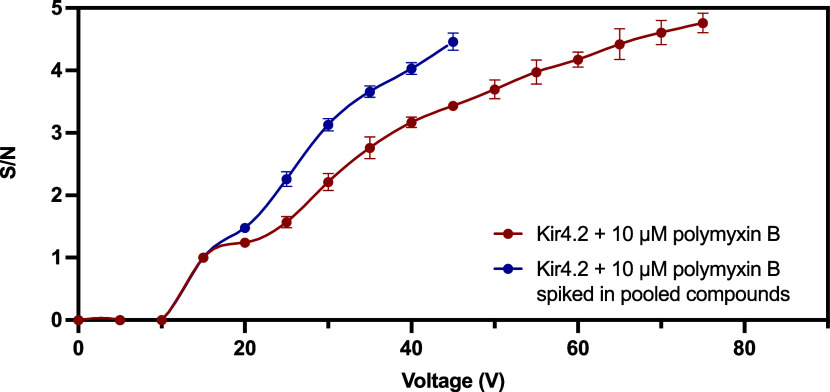
CIAS-MS voltage ramping for examining the dissociation of 10 μM Kir4.2 incubated with 10 μM polymyxin B alone or incubated with 10 μM polymyxin B spiked in a compound pool (each at 10 μM) at 21 voltages, ranging from 0 to 100 V with a 5 V interval. Red: Kir4.2 (10 μM) incubated with polymyxin B (10 μM); blue: Kir4.2 (10 μM) incubated with polymyxin B (10 μM) spiked in the pooled compounds (each at a concentration of 10 μM). The S/N ratio included all of the adduct peaks: [polymyxin B2 + H]^+^, [polymyxin B1 + H]^+^, [polymyxin B2 + K]^+^, and [polymyxin B1 + K]^+^.

The dissociation of the bound polymyxin B was observed only when the CID voltage reached 15 V or higher, for both Kir4.2 incubated with 10 μM polymyxin B and Kir4.2 incubated with 10 μM polymyxin B spiked in a pool of compounds (Figure 8). This is consistent with the expectation that the complex can only be ejected from the detergent micelle when a sufficient overall activation energy is reached. Notably, Kir4.2 incubated with 10 μM polymyxin B spiked in pooled compounds exhibited a more pronounced dissociation pattern with faster kinetics at lower CID voltages, in contrast to the Kir4.2 incubated with 10 μM polymyxin B only. The latter displayed a more gradual increase in dissociation as the CID voltage increased. Kir4.2 incubated with 10 μM polymyxin B only and with 10 μM polymyxin B spiked in pooled compounds were able to maintain packing of ions up to 75 and 45 V, respectively, with both samples stopped dissociating at 90 V (data not shown). In a system comprising multiple substances, such as proteins, ligands, water, buffer ions, and detergent, complex interactions occur. The extent of dissociation induced by the CID energy is influenced by various interactions and energy exchanges, including the stability of the noncovalent intermolecular interactions within the complex. The initial rapid increase of S/N ratio with increasing CID voltage at lower CID voltages and the subsequent slower dissociation at higher CID voltages in the spiked polymyxin B sample are likely attributable to the intricate interplay among these substances, resulting in the observed differences in dissociation pattern between the two samples. Additionally, the presence of additional components in the spiked sample contributed to higher background noise, which also accounted for the lower S/N ratio observed at the maximum voltage where it could maintain packing of ions, in comparison to the polymyxin B only sample. Nevertheless, the required CID voltage for dissociation to initiate and terminate for the same ligand at the same concentration in different mixtures remains consistent, highlighting the consistency of the current approach.

**Figure 9:**
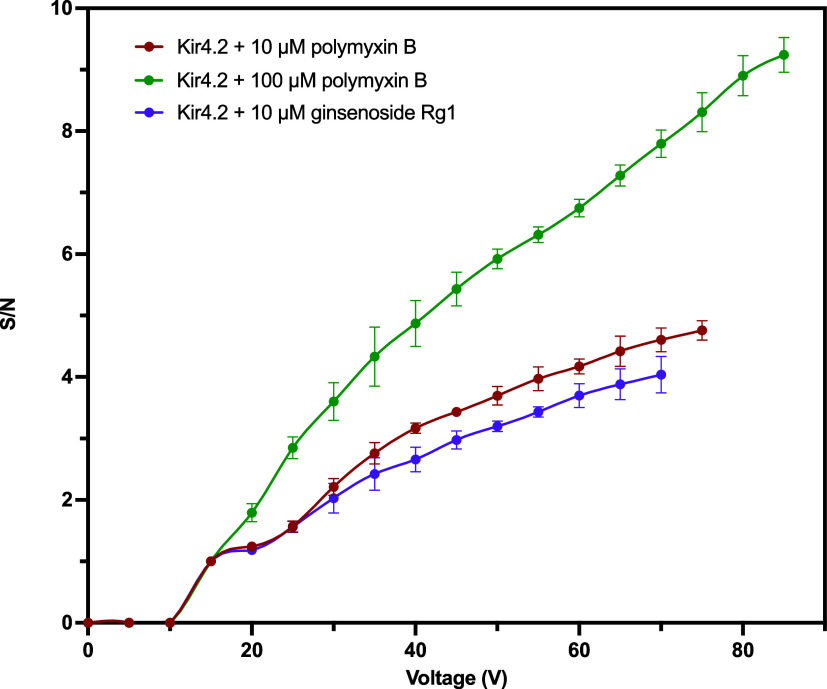
Comparison of CIAS-MS voltage ramping for ligand dissociation pattern of 10 and 100 μM polymyxin B, and 10 μM ginsenoside Rg1, incubated with 10 μM Kir4.2, at 21 voltages ranging from 0 to 100 V with a 5 V interval. Red: Kir4.2 incubated with 10 μM polymyxin B; green: Kir4.2 incubated with 100 μM polymyxin B; purple: Kir4.2 incubated with 10 μM ginsenoside Rg1. The S/N ratio included all adduct peaks: [polymyxin B2 + H]^+^, [polymyxin B1 + H]^+^, [polymyxin B2 + K]^+^, and [polymyxin B1 + K]^+^.

Next, Kir4.2 incubated with 10 and 100 μM polymyxin B was compared. In the case of the 100 μM polymyxin B sample, dissociation began at 15 V, similar to the 10 μM polymyxin B sample, but it was able to maintain packing of ions at a higher voltage of 85 V (Figure 9). Overall, the 100 μM polymyxin B sample obtained a higher S/N ratio at each voltage increment compared with the 10 μM polymyxin B. Notably, the S/N ratio of the 100 μM sample approximately doubled that of the 10 μM sample at most voltage increments, demonstrating a dose dependency (Figure S5). Kir4.2 incubated with 10 μM ginsenoside Rg1 exhibited a lower S/N ratio at each voltage increment compared to that of the 10 μM polymyxin B sample. The observed lower S/N for the ginsenoside Rg1 sample in comparison to the polymyxin B sample may suggest that ginsenoside Rg1 possesses a lower binding affinity than polymyxin B.

Overall, it was observed that the amount of bound ligand dissociated increased with the application of a higher CID energy. Polymyxin B demonstrated consistency in the CID voltages for both dissociation initiation and reaching a plateau, regardless of whether Kir4.2 was incubated exclusively with polymyxin B or with polymyxin B in a spiked pool. When Kir4.2 was incubated with polymyxin B at a 10-fold greater concentration, a higher S/N ratio was observed at each voltage, demonstrating a dose dependency.

## Conclusions

Functional studies have shown that polymyxin B induces cell depolarization in wild type but not in Kir4.2 knockout HK-2 cells with evidence of its cellular uptake.^16^ Molecular dynamics stimulation of Kir4.2 interacting with polymyxin B has also been reported.^16^ The observation of dissociated polymyxin B from a Kir4.2-polymyxin B complex was never reported previously. Using CIAS-MS, we were able to detect dissociated polymyxin B from a Kir4.2-ligand complex and to screen a natural product library comprising 2000 compounds. The liberation of membrane protein–ligand complexes within the detergent micelle was achieved by augmenting the overall activation energy of the MS system. The protein–ligand complex was captured by quadrupole mass selection, and the bound molecule was dissociated by CID. We explored and compared the dissociation behavior of the same ligand when incubated with the protein alone, when spiked into a mixture of pooled compounds, and at a 10-fold higher concentration using CID voltage ramps. The CID voltage dissociation curve revealed that the binding ligand at a 10-fold concentration showed a dose dependency.

CIAS-MS integrates all stages of affinity selection, including capture, separation, and dissociation within the mass spectrometer platform, offering a significant reduction in the overall time compared with hyphenated techniques. We successfully confirmed the physical interaction between the Kir4.2 channel and polymyxin B, which was previously shown to modify Kir4.2 channel functions by electrophysiological studies.^16^ Additionally, we identified a new binder for Kir4.2, ginsenoside Rg1.

## Methods

### Reagents and Compounds

Reagents for cell culture and biochemical experiments were purchased from Thermo Fisher Scientific and Sigma-Aldrich, unless stated otherwise.

### Cloning and Expression

The Kir4.2 DNA fragment was amplified through PCR using Phusion High-Fidelity DNA polymerase (New England Biolabs). The amplification was performed with a forward primer containing the *Kpn*I restriction site (5′- TAATTGGTACCGCCACCATGGATGCCATTAC ATC-3′) and a reverse primer containing the *Xba*I restriction site (5′- GCGCCGTCTAGAGCGACATTGCTCTGTTGTAATAAAAG −3′). The resulting PCR product was confirmed by gel electrophoresis and purified using the NucleoSpin Gel and PCR cleanup kit (MACHEREY-NAGEL). The purified PCR product was then double-digested with *Kpn*I/*Xba*I and ligated into the pEF6/V5-His vector, which carries a C-terminal V5 epitope (for detection using anti-V5 antibody) and a C-terminal polyhistidine (6 × His) sequence (for purification using nickel resin). Plasmid sequence was verified by DNA sequencing.

### Cell Culture

Expi293F cells (Thermo Fisher) were cultured in a ventilated 125 mL disposable shaker flask placed on an orbital shaker operating at 120 ± 5 rpm, maintaining a temperature of 37 °C and 8% CO_2_. The cells were cultured using Expi293 Expression Medium and routinely split every 3–4 days once the cell density reached 3–5 × 10^6^ viable cells/mL. Only cells exhibiting viability exceeding 95% and with a passage number lower than 25 were utilized for transfection.

### Transient Transfection

Transfections were conducted according to the manufacturer’s instruction with ExpiFectamine 293 Transfection Kit (ThermoFisher). Briefly, the cells were seeded at a density of 2.5 × 10^6^ viable cells/mL the day before transfection. On the day of transfection, the cells were diluted to a final density of 3 × 10^6^ viable cells/mL. Plasmid DNA (1.0 μg/mL of culture volume) diluted with Opti-MEM was complexed with 80 μL of ExpiFectamine293 diluted in Opti-MEM and transferred into the shaker flasks. Enhancer 1 and enhancer 2 were supplemented into the culture medium 20 h post-transfection. The transiently transfected cells were harvested 4 days post-transfection by centrifugation at 500*g* for 10 min and washed in 1 × DPBS (Thermo Fisher). The cell pellets were stored at −80 °C prior to purification.

### Kir4.2 Solubilization

Transfected cells were subjected to a washing step and subsequently resuspended in a solution containing 50 mM Tris base (pH 8), 150 mM NaCl, 1 mM PMSF, and 1× complete protease inhibitor cocktail (Roche, USA). The cells were lysed using a sonicator (BRANSON) with several short cycles of 10 s. The resulting cell lysate was then centrifuged at 15,000*g* at 4 °C for 30 min to pellet unlysed cells and cellular debris. A Sorvall WX Ultra Series ultracentrifuge (Thermo Fisher Scientific, Australia) was utilized to perform a further centrifugation step at 200,000*g* at 4 °C for 2 h. The supernatant from this step was removed, while membrane pellets were resuspended in solubilization buffer containing 50 mM Tris (pH 8), 150 mM NaCl, 10% glycerol, 1 mM PMSF, 1× complete protease inhibitor cocktail, and 1% DDM. The resulting solubilization mixture was subjected to agitation using a magnetic stir bar at 4 °C overnight. Following the overnight incubation, another round of centrifugation was performed at 200,000*g* at 4 °C for 1 h to pellet any remaining insolubilized material. The supernatant, containing the solubilized membrane protein, was carefully recovered for purification.

### Immobilized Metal Affinity Chromatography

Immobilized metal affinity chromatography (IMAC) was employed for Kir4.2 purification. The solubilized membrane proteins were supplemented with 10 mM imidazole prior to being loaded onto a HisPur Ni-NTA resin, which had been pre-equilibrated with two resin bed volumes of an equilibration buffer consisting of 50 mM Tris (pH 8), 150 mM NaCl, 10 mM imidazole, and 0.05% DDM. The binding of the target protein to the resin was carried out on a rotatory platform at 4 °C overnight. Subsequently, the unbound material was removed through centrifugation for 700*g*. To ensure efficient purification, the resin was washed four times with two resin bed volumes of a wash buffer comprising 50 mM Tris (pH 8), 150 mM NaCl, 25 mM imidazole, and 0.2% DDM. Finally, Kir4.2 was eluted from the resin in three consecutive steps, each employing one resin bed volume of an elution buffer containing 50 mM Tris, 150 mM NaCl, 400 mM imidazole, and 0.05% DDM. Samples (10 μL) from the wash steps and elution steps were collected for subsequent Western blot analysis and stored at 4 °C. The concentration of purified Kir4.2 was determined by A280 molar absorbance using a NanoDrop One spectrophotometer (Thermo Scientific).

### Western Blotting

Samples from solubilization and purification steps were mixed with 1× NuPAGE LDS sample buffer and 1× NuPAGE sample reducing agent. The mixture was heated at 50 °C for 10 min. SDS-polyacrylamide gel electrophoresis was performed using NuPAGE 4–12%, Bis-Tris gels. The gel was then transferred onto a Trans-Blot Turbo Mini 0.2 μm PVDF membrane (Bio-Rad). The membrane was blocked with 5% BSA in TBS-T (TBS + 0.1% Tween-20) for 1 h. Subsequently, the membrane was incubated with rabbit anti-V5 antibody (1:1000, Cell Signaling Technologies, D3H8Q) for 2 h at RT. Antibody binding was detected using IRDye 680RD-conjugated Goat Anti-Rabbit IgG H&L preabsorbed (1:10,000, Abcam, ab216777) for 1 h at RT. The protein bands were visualized using the Odyssey CLx Imaging System and quantified using ImageStudio software (Li-Cor).

### Compound Library

The compound library used was obtained from Compound Australia. The library contained 20 pools of DMSO with 100 compounds in each pool. Each pool was freeze-dried to remove the DMSO, and resuspended in 10 μL of methanol, prior to the incubation with 90 μL of protein. The final screening concentration for each compound in the pools was 10 μM.

### Protein Preparation for CIAS-MS

The three elutions of purified Kir4.2 proteins were passed through an Amicon Ultra-0.5 100-kDa molecular-weight cutoff (MWCO) concentrator (Millipore) to remove excess DDM micelle. The proteins were then subjected to buffer exchange into MS buffer containing 200 mM ammonium acetate (pH 8) with 2× CMC DDM using a NAP-5 column (Cytiva) prior to CIAS-MS experiments. For the CIAS-MS screening, 90 μL of the protein working solution at 10 μM was added to each compound library pool (dissolved in 10 μL of methanol) and incubated at RT for 30 min. Nsp9 protein was produced as previously described.^67^ Nsp9 was buffer-exchanged with 200 mM ammonium acetate (pH 7) using a NAP-5 column (Cytiva).

### Instrument Control and Data Acquisition

All experiments were conducted using a Bruker SolariX XR 12 T Fourier transform-ion cyclotron resonance mass spectrometer (Bruker Daltonics, Inc., Billerica, MA). The electrospray ionization (ESI) source was coupled with direct injection via a 500 μL Hamilton syringe equipped with the syringe pump. Data acquisition was performed using Solarix control software for a Bruker SolariX XR 12T in a Windows operating system.

### CIAS-MS

The direct injection was operated at a flow rate of 220 μL/h. Nitrogen gas was employed as the nebulizing gas with a pressure of 2 bar. The capillary voltage was set at 4000 V, and the end-plate voltage was set at −500 V. The dry gas flow rate was maintained at 4 L/min, and the temperature was set to 200 °C. The source optics, including the capillary exit, deflector plate, funnel 1, and skimmer 1, were set at voltages of 200, 220, 150, and 80 V, respectively. The quadrupole was configured to capture ions with a mass-to-charge ratio above 2500. Argon gas was employed as the collision gas, with 80% gas flow. A time-of-flight (TOF) of 0.65 ms was utilized. The collisional RF and frequency were kept at 1600 V_pp_ and 1 MHz, respectively. For CIAS-MS voltage experiments, the CID voltage was adjusted from 0 to 100 V with a 5 V interval. The observation window was set by the mass analyzer to detect only molecules with *m*/*z* below 2000.

### Native MS

Native MS analysis of nsp9 was conducted by using direct injection at a flow rate of 120 μL/h. The nebulizing gas pressure was maintained at 2 bar. The voltages for the capillary and end-plate offset were set to 4000 and −500 V, respectively. The dry gas flow rate and temperature were set to 4 L/min and 200 °C, respectively. The source optics capillary exit voltage and deflector plate voltage were adjusted to 200 and 220 V, respectively. Funnel 1 and skimmer 1 were operated with voltages of 150 and 30 V, respectively. The optical transfer frequency was set to 2 MHz, and the TOF was configured for 1.5 ms.

## Supporting Information Available

he Supporting Information is available free of charge on the ACS Publications Web site. The Supporting Information is available free of charge at https://pubs.acs.org/doi/10.1021/acschembio.3c00781.

Mass spectrum of 10 μM polymyxin B in 200 mM ammonium acetate with 10% methanol (Figure S1); CIAS-MS with CID enabled for Pool F alone and Ginsenoside Rg1 alone (Figure S2); native MS of nsp9 incubated with the library pool F (Figure S3); mass spectrum of 10 μM ginsenoside Rg1 in 200 mM ammonium acetate with 10% methanol (Figure S4); and CIAS-MS of Kir4.2 incubated with 10 and 100 μM polymyxin B with CID enabled under the same voltage of 60 V (Figure S5) (PDF)
