# Supplementary material for: Advancing Kir4.2 Channel Ligand Identification through Collision-Induced Affinity Selection Mass Spectrometry
Source: ACS Chem Biol. 2024 Mar 7;19(3):763–73. doi: 10.1021/acschembio.3c00781 (PMC10949200; doi:10.1021/acschembio.3c00781)
Supplement: Supplementary file 1 — cb3c00781_si_001.pdf [file cb3c00781_si_001.pdf]

Supporting information

Advancing Kir4.2 Channel Ligand Identification through Collision-Induced Affinity Selection Mass Spectrometry

Yushu Gu[1], Miaomiao Liu[1], Linlin Ma[1], Ronald J. Quinn[1]*

[1]Griffith Institute for Drug Discovery, Griffith University, Brisbane, Queensland, Australia

[2]School of Environment and Science, Griffith University, Brisbane, Queensland, Australia

***Corresponding Author**
r.quinn@griffith.edu.au

SUPPLEMENTARY FIGURE

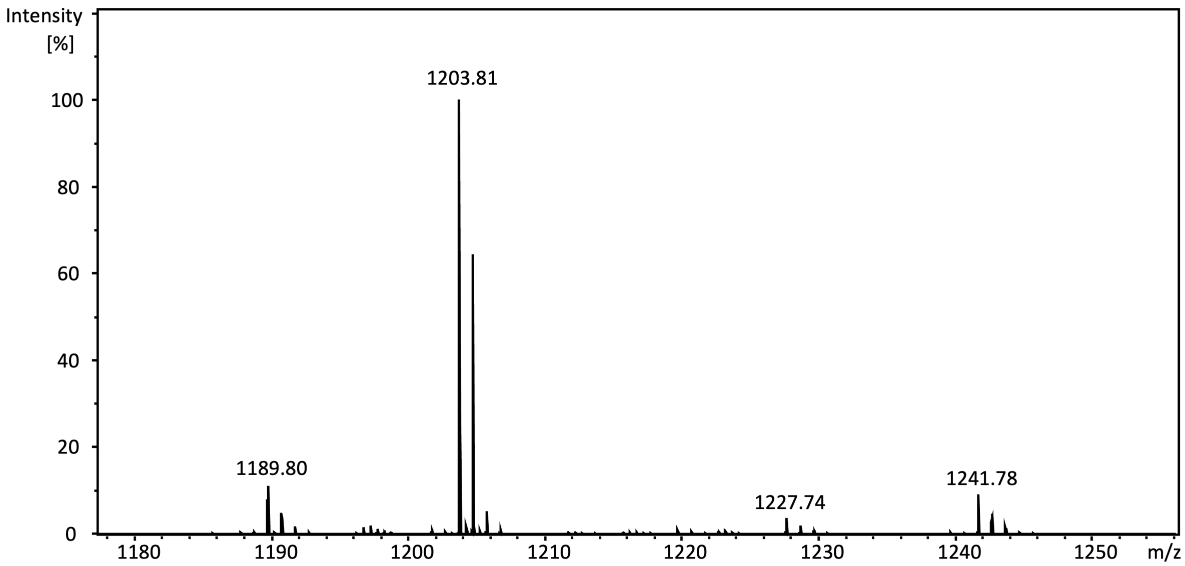

Figure S1. Mass spectrum of 10 µM polymyxin B in 200 mM ammonium acetate with 10% methanol. The base peak for polymyxin B2 were observed at 1189.80 *m/z* with a potassium adduct at 1227.74 *m/z*, comprising 34% of the base peak intensity. For polymyxin B1, the base peak was observed at 1203.81 *m/z*, with a potassium adduct at 1241.78 *m/z,* accounting for 9% of the base peak intensity.

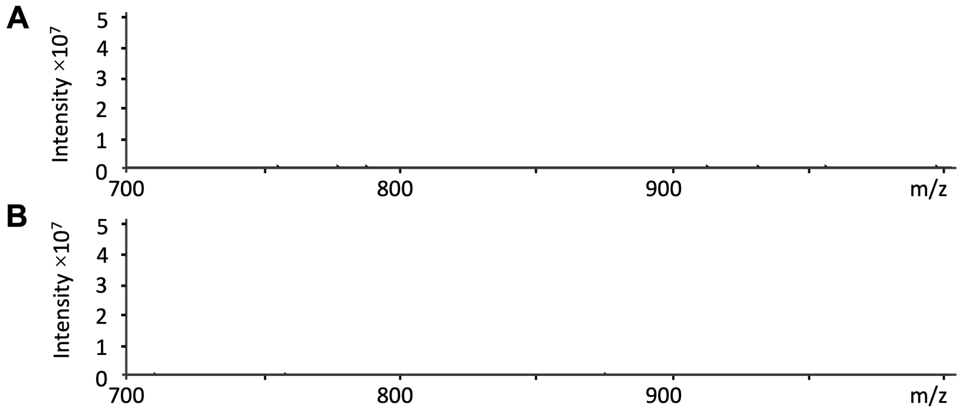

Figure S2. CIAS-MS with CID enabled for (**A**) Pool F alone and (**B**) Ginsenoside Rg1 alone.

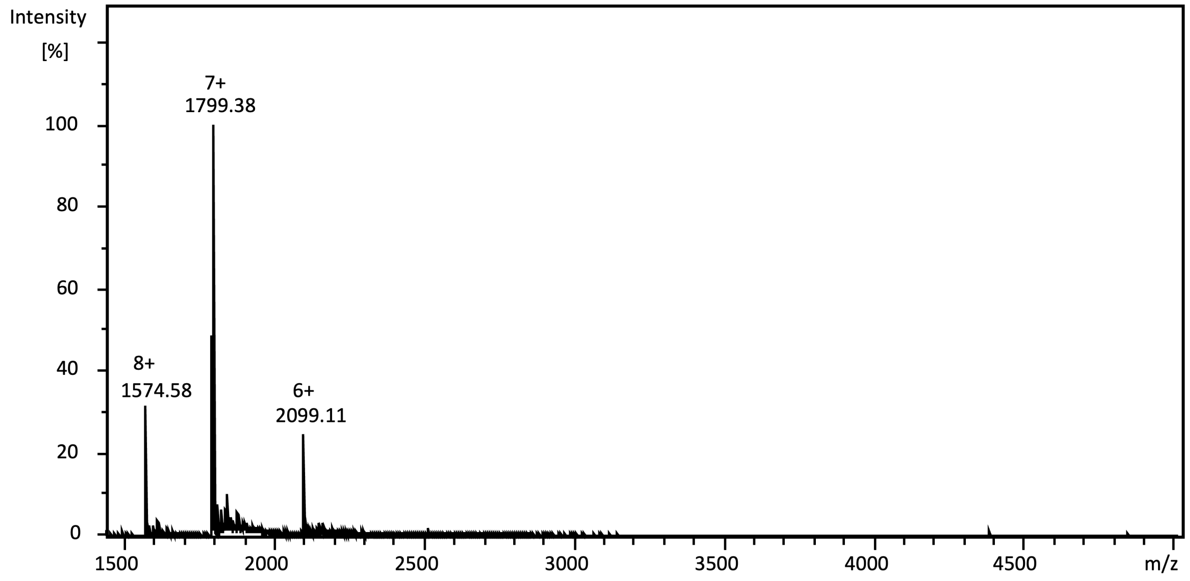

Figure S3. Native MS of nsp9 incubated with the library pool F that produced the Kir4.2 hit. 90 µL of nsp9 (10 µM) in 200 mM ammonium acetate, pH 7.0, was added to the library pool dissolved in 10 µL methanol.

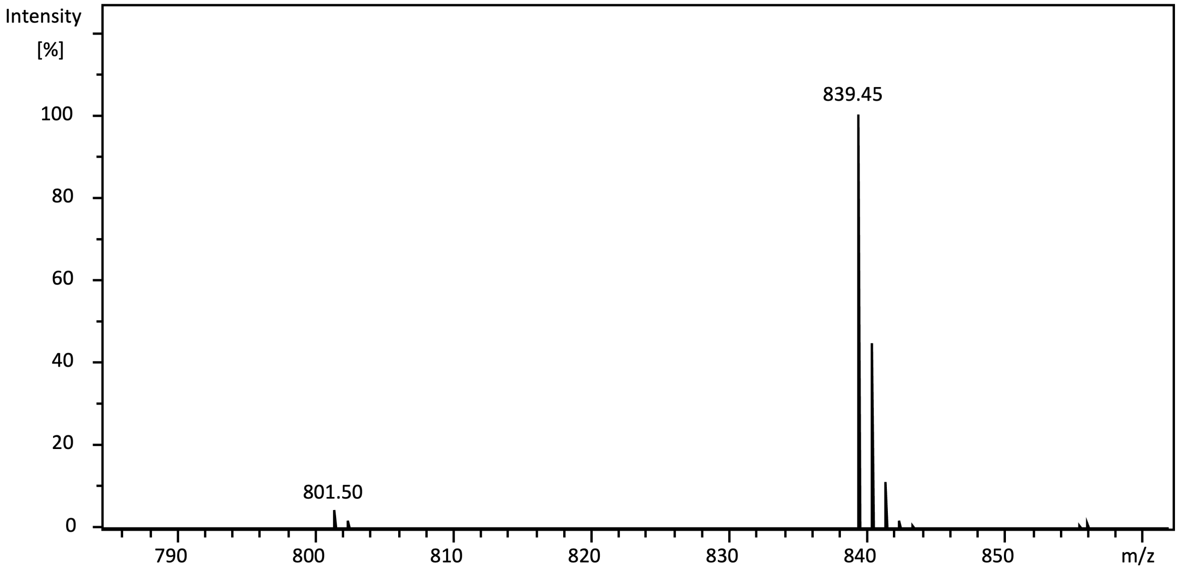

Figure S4. Mass spectrum of 10 µM ginsenoside Rg1 in 200 mM ammonium acetate with 10% methanol. Under positive-mode ESI condition, ginsenoside Rg1 was observed as a base peak of 839.45 *m/z* (potassium adduct) and a 5% hydrogen adduct 801.50 *m/z*.

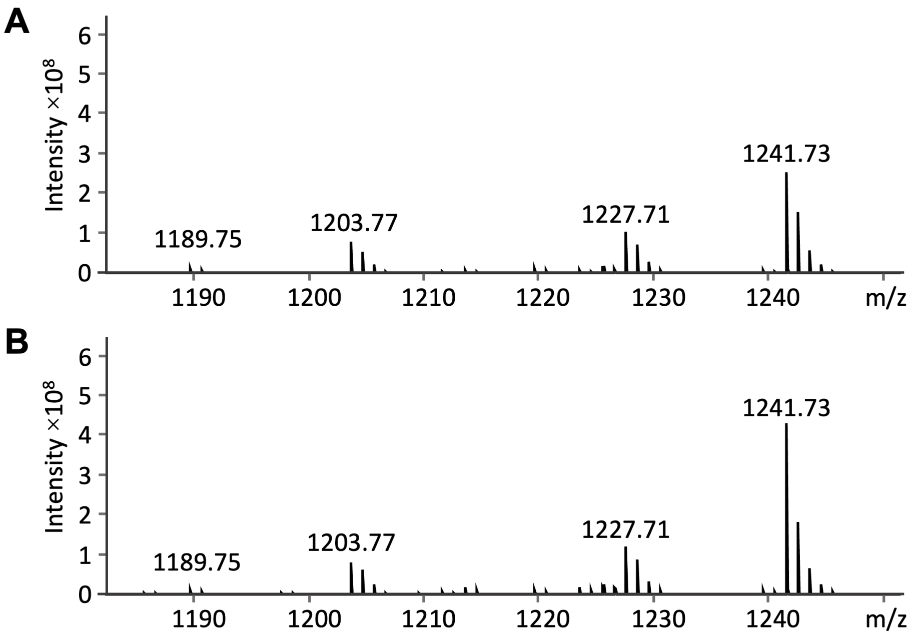

Figure S5. CIAS-MS of Kir4.2 incubated with (**A**) 10 µM polymyxin B and (**B**) 100 µM polymyxin B with CID enabled under the same voltage of 60 V.
